# Detection of Anti-SARS-CoV-2-S2 IgG Is More Sensitive Than Anti-RBD IgG in Identifying Asymptomatic COVID-19 Patients

**DOI:** 10.3389/fimmu.2021.724763

**Published:** 2021-08-19

**Authors:** Baolin Liao, Zhao Chen, Peiyan Zheng, Linghua Li, Jianfen Zhuo, Fang Li, Suxiang Li, Dingbin Chen, Chunyan Wen, Weiping Cai, Shanhui Wu, Yanhong Tang, Linwei Duan, Peilan Wei, Fangli Chen, Jinwei Yuan, Jinghong Yang, Jiaxin Feng, Jingxian Zhao, Jincun Zhao, Baoqing Sun, Airu Zhu, Yimin Li, Xiaoping Tang

**Affiliations:** ^1^Guangzhou Eighth People’s Hospital, Guangzhou Medical University, Guangzhou, China; ^2^State Key Laboratory of Respiratory Disease, National Clinical Research Center for Respiratory Disease, Guangzhou Institute of Respiratory Health, The First Affiliated Hospital of Guangzhou Medical University, Guangzhou, China; ^3^Department of Allergy and Clinical Immunology, State Key Laboratory of Respiratory Disease, National Clinical Research Center for Respiratory Disease, National Center for Respiratory Medicine, Guangzhou Institute of Respiratory Health/The First Affiliated Hospital of Guangzhou Medical University, Guangzhou, China

**Keywords:** SARS-CoV-2, COVID-19, asymptomatic infections, IgG, antibody response, neutralizing antibody

## Abstract

Characterizing the serologic features of asymptomatic SARS-CoV-2 infection is imperative to improve diagnostics and control of SARS-CoV-2 transmission. In this study, we evaluated the antibody profiles in 272 plasma samples collected from 59 COVID-19 patients, consisting of 18 asymptomatic patients, 33 mildly ill patients and 8 severely ill patients. We measured the IgG against five viral structural proteins, different isotypes of immunoglobulins against the Receptor Binding Domain (RBD) protein, and neutralizing antibodies. The results showed that the overall antibody response was lower in asymptomatic infections than in symptomatic infections throughout the disease course. In contrast to symptomatic patients, asymptomatic patients showed a dominant IgG-response towards the RBD protein, but not IgM and IgA. Neutralizing antibody titers had linear correlations with IgA/IgM/IgG levels against SARS-CoV-2-RBD, as well as with IgG levels against multiple SARS-CoV-2 structural proteins, especially with anti-RBD or anti-S2 IgG. In addition, the sensitivity of anti-S2-IgG is better in identifying asymptomatic infections at early time post infection compared to anti-RBD-IgG. These data suggest that asymptomatic infections elicit weaker antibody responses, and primarily induce IgG antibody responses rather than IgA or IgM antibody responses. Detection of IgG against the S2 protein could supplement nucleic acid testing to identify asymptomatic patients. This study provides an antibody detection scheme for asymptomatic infections, which may contribute to epidemic prevention and control.

## Introduction

COVID-19, caused by SARS-CoV-2 infection, has caused more than 100 million laboratory-confirmed infections and more than 2 million deaths so far. The number of patients is increasing at an alarming rate of hundreds of thousands every day, resulting in a heavy medical and economic burden to the world (1–3).

Since the outbreak of COVID-19, asymptomatic infection has been reported (4). Asymptomatic individuals do not show clinical symptoms, and are usually identified through mass-community screening or contact tracing (5), making them a likely population that contributes to the continuous community spread of SARS-CoV-2 virus. Some studies even believe that asymptomatic individuals are more infectious than those with symptoms (6). Recently, a systematic review showed that asymptomatic infections accounted for at least one-third of SARS-CoV-2 infections (7), further indicating that asymptomatic infections may play a pivotal role in the COVID-19 pandemic (8). Thereby, management of asymptomatic infections has become one of the key measures to control the COVID-19 pandemic (9). Detection of viral nucleic acid by RT-qPCR is considered the gold standard to diagnose SARS-CoV-2 infections (10). However, this approach is limited by the influence of sampling time, types of clinical specimens and method of inactivation, which can yield false-negative results and lead to misdiagnosis, especially in asymptomatic infections (11–13). Detection of SARS-CoV-2-specific antibodies is an important complementary method for the diagnosis of COVID-19 (14). There is an urgent need for a more sensitive antibody detection scheme of asymptomatic infections.

Here, we conducted a study to compare the antibody profile of asymptomatic infections to patients with different disease severity. We also compared the pros and cons of different antibody detection schemes for the diagnosis of asymptomatic infections. We found that asymptomatic infections elicit weaker antibody responses than symptomatic infections, and primarily induced IgG-based antibody responses throughout the disease course. Remarkably, detection of anti-SARS-CoV-2-S2 IgG is more sensitive than anti-RBD IgG in identifying asymptomatic COVID-19 patients.

## Materials and Methods

### Patient Enrollment and Sample Collection

From January to April, 2020, 59 SARS-CoV-2 infected patients, confirmed by real-time PCR and hospitalized in the Guangzhou Eighth People’s Hospital, were enrolled in this study. The patients were categorized into 3 groups based on their disease severity, including 18 asymptomatic patients, 33 mild-ill patients and 8 severe patients. Plasma samples were collected from each patient at multiple time points. Clinical data including patient’s demographic information and clinical outcome was retrieved from the medical records. Plasma samples from eight healthy donors collected in 2017-2018 were used as controls in this study.

### Detection of Anti-SARS-CoV-2-RBD IgA, IgM and IgG by Electrochemiluminescence

The Kaeser 6600 automatic chemiluminescence immunoanalyzer and the matching reagents kit (Guangzhou Kangrun Biotech Co. Ltd, Guangzhou, China) was used to detect the SARS-CoV-2-specific IgM, IgA and IgG levels using a two-step indirect detection method as described previously (15). Briefly, the amino group on the RBD protein was coupled with the carboxyl group of the magnetic beads, and the antigen was fixed to form RBD-coating magnetic beads. The SARS-CoV-2 RBD-specific antibodies in the testing serum could bind to the RBD-coating magnetic beads, and acridine ester derivatives-coupled anti-human IgA, IgM and IgG antibodies were added. After the unbound substances were removed, a photomultiplier was used to detect light signals from acridine ester that were converted to obtain the corresponding signal value. The relative light signal values, expressed in relative light units (RLU), indicated the specific IgM, IgA and IgG levels. The relative light signal value is equivalent to the original signal value over the specific antibody cut-off value. The cut-off values of IgM, IgA and IgG were 11 300, 56 492 and 42 213, respectively. A relative luminescence value (RLV) greater than or equal to 1.0 was considered positive for SARS-CoV-2 RBD specific IgM, IgA and IgG.

### Comparison of Antibody Response Against Different SARS-CoV-2 Proteins

To assess the antibody response against the different SARS-CoV-2 proteins or different fragments of the spike protein, SARS-CoV-2 S (spike protein, 1203 aa), S1 (675 aa), S2 (533 aa), RBD (228 aa) and N (424 aa) proteins were obtained from Sino Biological, Inc (Beijing) and used as coating antigens in our in-house ELISA to detect the antigen-specific IgG. Briefly, 96-well plates (Jet, Biofil Co., Ltd, Guangzhou) were coated with 100 μl/well (0.5 μg/ml) of SARS-CoV-2 S, S1, S2, RBD or N protein in DPBS buffer (Thermo Fisher Scientific (China), Shanghai) overnight at 4°C. After blocking (DPBS, 10%FBS), 100 μl of diluted plasma (1:100) were added and plates were incubated at 37°C for one hour. After washing, plates were incubated with 100 μl of HRP-conjugated mouse anti-human IgG (H+L) antibody (Catalog No: 109-035-088, Jackson ImmunoResearch, West Grove, PA) at 37°C for one hour. Reactions were visualized by adding 50 μl of TMB (3,3’,5,5’-Tetramethylbenzidine) substrate solution (Biohao Biotechnology Co., Ltd., Beijing). Each plate was monitored to have the same reaction time and the optical densities at 450 nm were then read. The mean value of the healthy donor plasma (named HD group) collected in 2017-2018 plus 3 standard deviations was used as the detection threshold.

### Focus Reduction Neutralization Test

SARS-CoV-2 focus reduction neutralization test (FRNT) was performed in a certified Biosafety Level 3 lab. Fifty microliters of plasma samples were serially diluted, mixed with 50 μl of SARS-CoV-2 virus (100 focus forming unit, FFU) in 96-well plates and incubated for 1 hour at 37°C. The mixtures were then transferred to 96-well plates seeded with Vero E6 cells (ATCC, Manassas, VA) and incubated for 1 hour at 37°C to allow virus entry. Inoculums were then removed before adding the overlay media (100 μl MEM containing 1.2% Carboxymethylcellulose, CMC). The plates were then incubated at 37°C for 24 hours. Overlays were removed and cells were fixed with 4% paraformaldehyde solution for 30 min. Cells were permeabilized with 0.2% Triton X-100 and incubated with cross-reactive rabbit anti-SARS-CoV-N IgG (Cat: 40143-R001, Sino Biological, Inc, Beijing) for 1 hour at 37°C before adding HRP-conjugated goat anti-rabbit IgG(H+L) antibody (1:4000 dilution) (Catalog Number: 111-035-144, Jackson ImmunoResearch, West Grove, PA). Cells were further incubated at 37°C. The reactions were developed with KPL TrueBlue Peroxidase substrates (Seracare Life Sciences Inc, Milford, MA). The numbers of SARS-CoV-2 foci were calculated using an Elispot reader (Cellular Technology Ltd, Shaker Heights, OH).

### The Coincidence Between Neutralizing Antibodies and Binding Antibodies

In order to evaluate the predictive value of the binding antibodies for the neutralizing antibodies, we calculated the true positive rate (TPR), false positive rate (FPR), true negative rate (TNR), false negative rate (FNR), positive predictive value (PPV), negative predictive value (NPV), false discovery rate (FDR), and overall accuracy (OA) during the evaluation. The calculation formula is as follows:

TPR=TP/(TP+FP)

FPR=FP/(FP+TN)

TNR=TN/(TN+FP)

FNR=FN/(TP+FN)

PPV=TP/(TP+FP)

NPV=TN/(TN+FN)

FDR=FP/(TP+FP)

OA=(TP+TN)/(TP+FP+FN+TN)

### Statistical Analysis

Statistical analysis was performed using Graphpad Prism software, version 7.00. Shapiro-Wilk normality test was used to evaluate the type of data distribution. One-way ANOVA with Holm-Sidak's multiple comparisons test or Friedman test with Dunn's multiple comparisons test was used for comparing the IgA/IgM/IgG concentration-time curve of the three groups. Nonlinear regression was used to map the trend of antibodies or positive detection sensitivities of antibodies over time. Linear regression and Spearman correlation coefficient were used to assess the relationship between binding antibodies and neutralizing antibody. A p-value ≤0.05 was considered statistically significant (*p-values of ≤0.05. **p-values of ≤0.01. ***p- values of ≤0.001. ****p-values of ≤0.0001). All values are depicted as mean ± standard error of measurement (SEM).

### Ethics Statement

This study was conducted according to the ethical principles of the Declaration of Helsinki. Ethical approval was obtained from the Ethics Committee of Guangzhou Eighth People’s Hospital (202002135). Written informed consent was obtained from all participants.

## Results

### Patients and Clinical Information

Fifty-nine SARS-CoV-2-infected patients, confirmed by real-time qPCR and hospitalized in the Guangzhou Eighth People’s Hospital, were enrolled in this study. Plasma samples were collected from each patient at multiple time points to analyze the kinetics of antibody responses, and a total of 272 plasma samples were finally collected. As shown in **Table 1**, 18 asymptomatic patients (AP), 33 mildly ill patients (MP) and 8 severely ill patients (SP) were enrolled in this study. The mean age was 31.8 ± 15.5 years for AP, 47.3 ± 14.6 years for MP, 58.5 ± 9.62 years for SP. All patients were eventually cured and discharged from the hospital. Because asymptomatic infections do not develop symptoms, the day that the nucleic acid test was first positive was defined as day 0 post-onset.

**Table 1:** Patients involved in this study.

Group		Asymptomatic Patients	Mildly ill Patients	Severely ill Patients
Numbers		18	33	8
Age (years old)	$\bar{X}$ ± *SD*	31.8 ± 15.5	47.3 ± 14.6*	58.5 ± 9.62*
Sex				
Male		7	17	7
Female		11	16	1
Outcome				
Discharge		18	33	8
Death		0	0	0
Virus shedding time (days)	*median* ± *IQR*	3.0 ± 4.5	19.0 ± 15.5*	18.0 ± 27.5
Duration of hospitalization (days)	*median* ± *IQR*	8.0 ± 5.0	21.0 ± 10.0*	26.5 ± 14.3*

$\bar{X}$ mean; SD, standard deviation; IQR, interquartile range; *P < 0.05 (*vs.* asymptomatic patients, Mann-Whitney test).

### The Kinetics of Anti-SARS-CoV-2-RBD IgA/IgM/IgG Were Different Among AP, MP and SP

To understand the kinetics of antibody responses against SARS-CoV-2 in patients, plasma IgA, IgM and IgG against the SARS-CoV-2 receptor binding domain (RBD) were detected by electrochemiluminescence (ECL).

**Figure 1:**
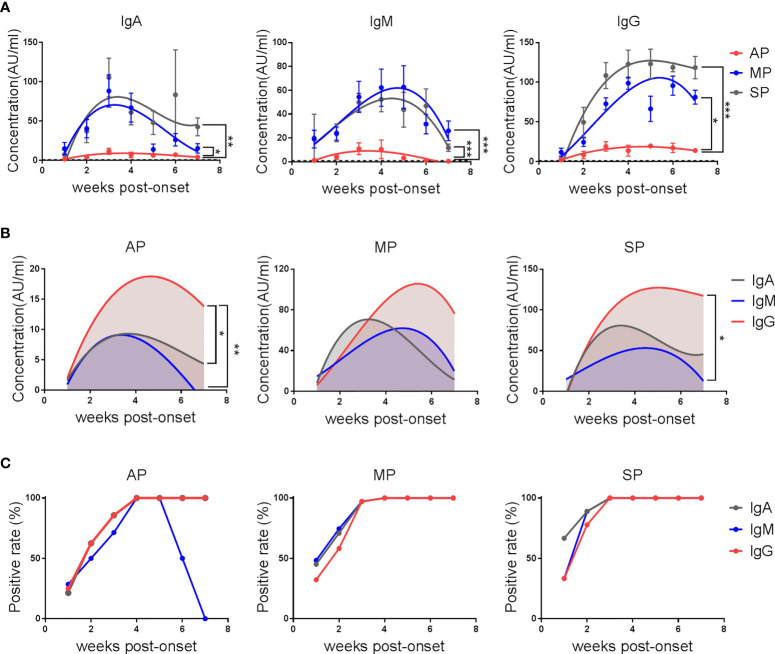
The kinetics of anti-SARS-CoV-2-RBD IgA/IgM/IgG in COVID-19 patients. Serum IgA, IgM and IgG against the RBD protein of SARS-CoV-2 were detected by electrochemiluminescence. **(A)** The kinetics of anti-SARS-CoV-2-RBD IgA/IgM/IgG antibodies in AP, MP and SP groups. The line is a trend line fitted by Nonlinear Regression method. Antibody concentrations were expressed as mean ± SEM. **(B)** The kinetics of different anti-SARS-CoV-2-RBD antibody isotypes in each group. The line is a trend line fitted using the nonlinear regression method. **(C)** Positive rates of different anti-SARS-CoV-2-RBD isotypes over time in each group. One-way ANOVA with Holm-Sidak’s multiple comparisons test was used. A p-value of ≤ 0.05 was considered statistically significant (*p-values of ≤ 0.05; **p-values of ≤ 0.01; ***p-values of ≤ 0.001).

As shown in **Figure 1A**, the average IgA, IgM and IgG responses in MP and SP were much more robust than in AP. The kinetics of IgA antibodies in the three groups of patients showed a similar trend, beginning to rise in the first week after disease onset, reaching a peak in the third week, and starting to decline from the fourth week. In the seventh week, reduced but still detectable IgA against SARS-CoV-2 RBD was observed in each group. The concentrations of IgA reached similar peak values in MP and SP, but decreased faster in MP. Unlike IgA, the IgM response peaked in the third week for AP, and in the fourth to fifth week for MP and SP, with a very close dynamics between MP and SP. As for the IgG response, the average anti-RBD IgG concentrations of AP and SP peaked in the fourth week and plateaued till the end point of this study. However, the IgG level in MP did not reach its peak until the end of the fifth week, which was about 2 weeks later than the other two groups, and no plateau was observed. These results suggest that SARS-CoV-2 can only elicit a limited anti-RBD antibody response in asymptomatic infections.

Further analysis of the antibody isotypes against RBD in the plasma of patients revealed that anti-RBD IgG was always the dominant type of antibody in AP. The levels of IgA and IgM were similar by the fourth week of admission, but IgM declined faster than IgA and became undetectable within week 7 (**Figure 1B**). In MP, anti- SARS-CoV-2-RBD antibodies were dominated by IgM in the first week, IgA in the second and third weeks, and IgG after the third week. From the fourth week, the IgA level fell below the IgM level. In SP, the antibodies were dominated by IgM in the first week, IgA and IgG in the second week, and IgG from the third week on.

Consistent with the much weaker antibody responses in AP, the percent positive rates of SARS-CoV-2 RBD-specific IgA, IgM and IgG were lower in AP, with the positive rates of all these three isotypes reaching 100% by the end of the third week in MP and SP but not in AP. Specifically, the positive rate of IgM dropped rapidly from the sixth week and became 0 by the end of the 7th week in AP (**Figure 1C**).

These results demonstrate that the kinetics and magnitude of the antibody responses in patients with different disease severity are different. More importantly, detecting anti-RBD IgG rather than IgM and IgA antibodies, even at early stage after infection, can be helpful for identifying AP.

### The Kinetics of IgG Response Against SARS-CoV-2-RBD/S1/S2/S/N Varied Among AP, MP and SP

**Figure 2:**
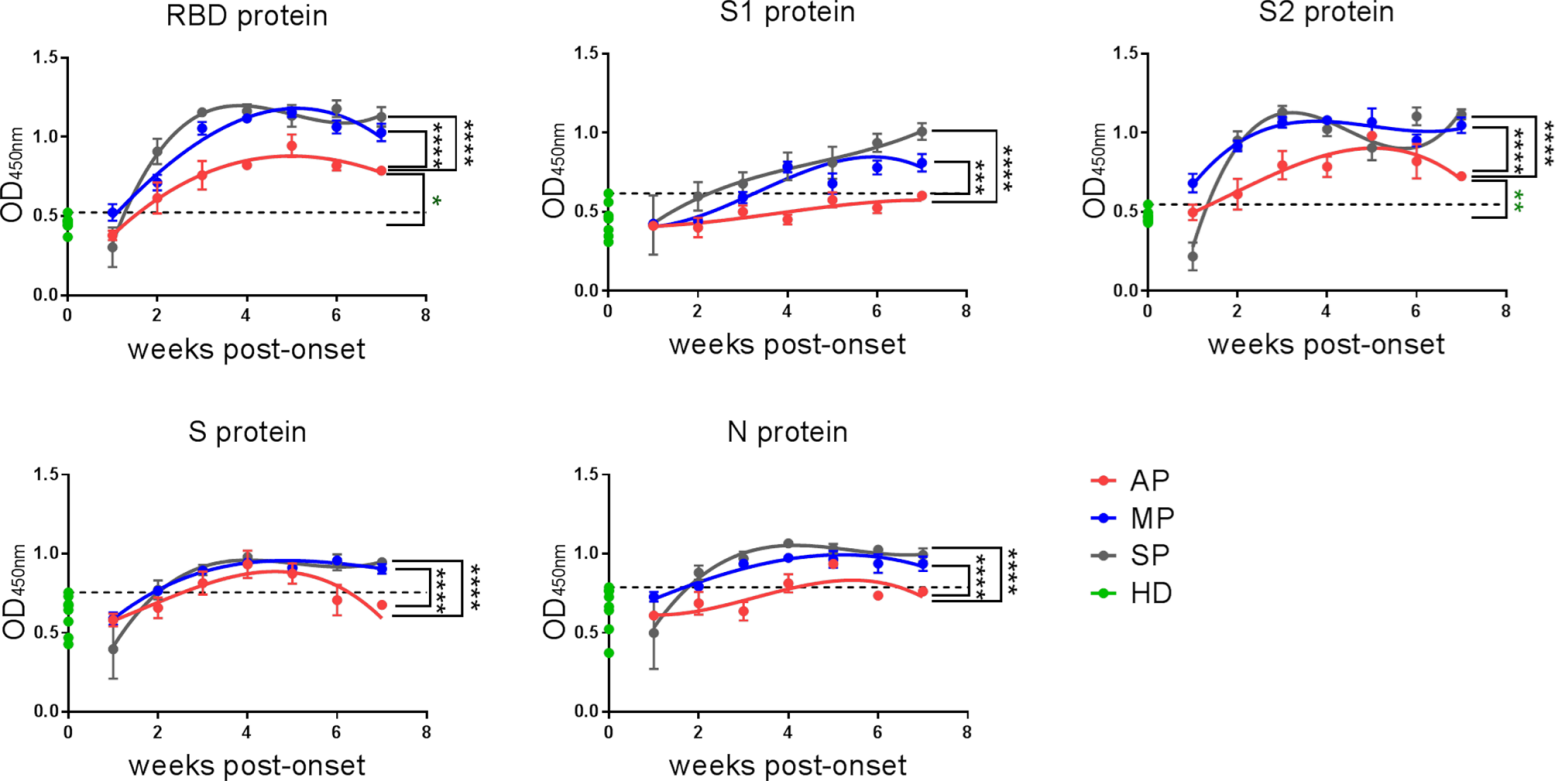
The kinetics of IgG against SARS-CoV-2-RBD/S1/S2/S/N of COVID-19 patients. IgG against RBD/S1/S2/S/N proteins of SARS-CoV-2 in plasma were detected by ELISA. The line is a trend line fitted by Nonlinear Regression method. OD values were expressed as mean ± SEM. Eight healthy donor plasma samples collected in 2017-2018 served as a control group (HD group) in this experiment. Threshold (dashed line) was defined as mean (of HD group) + 3SD. One-way ANOVA with Holm-Sidak’s multiple comparisons test was used. A p-value of ≤ 0.05 was considered statistically significant (*p-values of ≤ 0.05; **p-values of ≤ 0.01; ***p-values of ≤ 0.001; ****p-values of ≤ 0.0001).

In order to further dissect which structural proteins of SARS-CoV-2 are mainly targeted by the IgG antibodies and to understand their kinetics, we used ELISA to detect the antigen-specific IgG recognizing the different SARS-CoV-2 proteins. We included RBD (aa 319-514 of the spike protein), S1 (aa 1-685 of the spike protein), S2 (aa 686-1213 of the spike protein), S (aa 1-1213 of the spike protein), and N (aa 1-419 of the nucleocapsid protein) (**Figure 2**). The kinetics of anti-RBD IgG detected by ELISA (**Figure 2**) was consistent with that detected by the ECL assay (**Figure 1A**). Surprisingly, although the S1 protein contains the RBD fragment, the anti-S1 IgG kinetics were quite different from those of anti-RBD IgG, with a concordant upward trend in all the three groups. While the anti-S2 IgG response peaked in the third week post-onset in both MP and SP, it peaked in the fifth week in AP.

Overall, assays against the RBD and S2 proteins gave better signal and higher positive rates for all the three groups, and the IgG responses against all the indicated SARS-CoV-2 structural proteins were much weaker in AP than in MP or SP. Taken together, RBD and S2, but not other proteins (S1, S and N) are suitable targets for detecting SARS-CoV-2-reactive IgG antibodies in AP.

### The Kinetics of Neutralizing Antibodies and Their Correlation With Binding Antibodies

**Figure 3:**
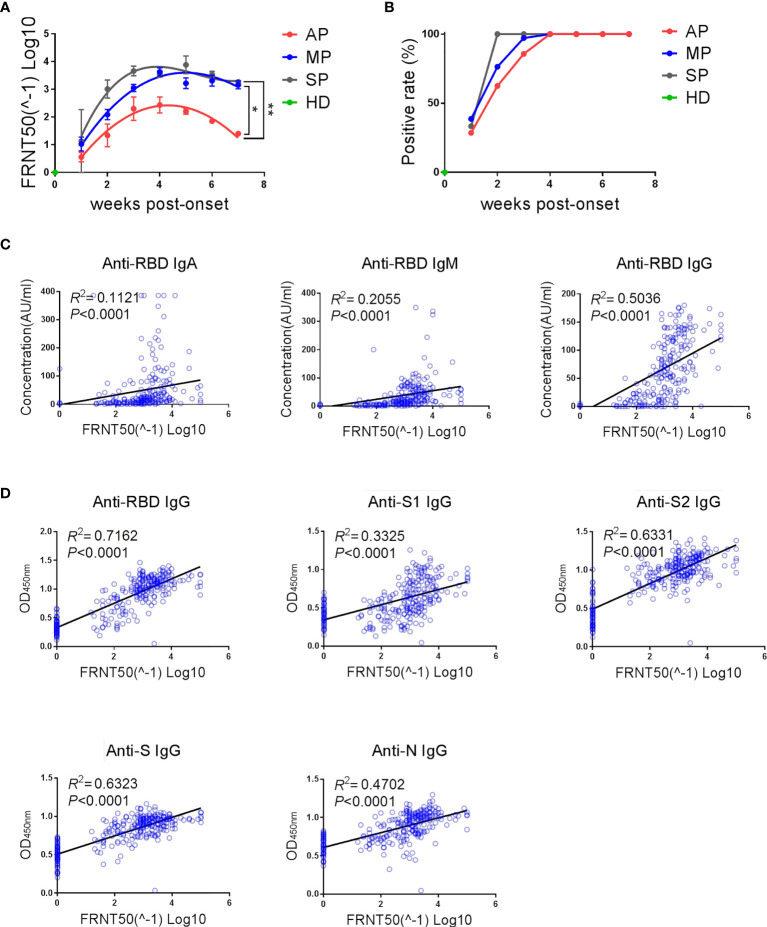
The kinetics of neutralizing antibodies and its strong correlation with the magnitude of anti-SARS-CoV-2 binding antibodies. **(A)** Kinetics of neutralizing antibodies in different groups. The line is a trend line fitted by Nonlinear Regression method. The neutralizing antibody titers were expressed as mean ± SEM. **(B)** Positive rates of neutralizing antibodies over time in different groups. **(A, B)** One-way ANOVA with Holm-Sidak’s multiple comparisons test was used. A p-value of ≤ 0.05 was considered statistically significant (*p-values of ≤ 0.05; **p-values of ≤ 0.01). **(C)** Correlation between neutralizing antibody titers and RBD-specific IgA/IgM/IgG concentrations detected by ECL assay was analyzed by linear regression. **(D)** Correlation between neutralizing antibody titers and multiple structural protein (RBD/S1/S2/S/N)-specific IgG levels detected by ELISA assay was analyzed by linear regression.

In the previous assays, we have evaluated the SARS-CoV-2-reactive IgG antibodies that possess binding activity, but not their neutralization capacity. Here, neutralizing antibodies in the different groups of patients were assessed using authentic SARS-CoV-2 virus in a focus reduction neutralization test (FRNT). As shown in **Figure 3A**, the dynamics of neutralizing antibodies in the three groups was similar to those of anti-RBD IgG (**Figure 1A**), which is in line with previous studies demonstrating that neutralizing antibodies are mainly IgG antibodies that bind to the RBD region (16). The magnitude of neutralizing antibody response was high in SP, moderate in MP, and low in AP (**Figure 3A**). Further, the positive rate of neutralizing antibodies reached 100% two weeks faster in SP than in MP or AP (**Figure 3B**).

In order to investigate the association between binding antibodies and neutralizing antibodies, we performed correlation analysis and linear regression analysis on the levels of neutralizing antibodies and binding antibodies (**Figures 3C, D**). As shown in **Figures 3C, D**, the titers of neutralizing antibodies (FRNT 50) had a significant linear correlation with the concentration of IgA/IgM/IgG against SARS-CoV-2-RBD (**Figure 3C**) and with the level of IgG antibodies against multiple SARS-CoV-2 structural proteins (**Figure 3D**). To further explore whether the presence of neutralizing antibodies can be predicted by detection of binding antibodies, the consistency between the detection results of different binding antibodies and the neutralizing antibodies was analyzed (**Table 2**). As expected, there was indeed a certain degree of consistency between the presence of neutralizing antibodies and binding antibodies, although this degree depended on the type of binding antibody. Among the binding antibodies, different isotypes of anti-RBD antibodies detected by ECL and anti-S2 or anti-RBD IgG detected by ELISA showed an overall accuracy (OA) of over 90% in predicting the presence of neutralizing antibodies. Specifically, anti-S2 IgG had the highest overall accuracy (94.85%) and highest negative prediction value (97.87%), indicating that when anti-S2-IgG is negative, the neutralizing antibody is most likely to be negative. Whereas anti-RBD-IgG detected by ELISA had the highest positive prediction value (98.48%), indicating that when anti-RBD-IgG is positive, the neutralizing antibody is most likely to be positive. In line with this, the linear regression results showed that the linear fit between RBD-IgG and neutralizing antibody detected by ELISA assay was the best (*R^2^ = *0.7162, *P*<0.0001) (**Figure 3D**).

**Table 2:** Overall performance of binding antibodies for predicting neutralizing antibodies.

Method	Binding antibody		Neutralizing antibody		TPR(%)	FPR(%)	FNR(%)	TNR(%)	PPV(%)	NPV(%)	FDR(%)	OA(%)
			Positive	Negative								
ECL	Anti-RBD IgA	Positive	202	7	94.84	11.86	5.16	88.14	96.65	82.54	3.35	93.38
		Negative	11	52								
	Anti-RBD IgM	Positive	203	6	95.31	10.17	4.69	89.83	97.13	84.13	2.87	94.12
		Negative	10	53								
	Anti-RBD IgG	Positive	194	3	91.08	5.08	8.92	94.92	98.48	74.67	1.52	91.91
		Negative	19	56								
ELISA	Anti-RBD IgG	Positive	195	3	91.55	5.08	8.45	94.92	98.48	75.68	1.52	92.28
		Negative	18	56								
	Anti-S1 IgG	Positive	107	4	50.23	6.78	49.77	93.22	96.40	34.16	3.60	59.56
		Negative	106	55								
	Anti-S2 IgG	Positive	212	13	99.53	22.03	0.47	77.97	94.22	97.87	5.78	94.85
		Negative	1	46								
	Anti-S IgG	Positive	177	0	83.10	0.00	16.90	100.00	100.00	62.11	0.00	86.76
		Negative	36	59								
	Anti-N IgG	Positive	155	4	72.77	6.78	27.23	93.22	97.48	48.67	2.52	77.21
		Negative	58	55								

TPR, true positive rate; FPR, false positive rate; TNR, true negative rate; FNR, false negative rate; PPV, positive predictive value; NPV, negative predictive value; FDR, false discovery rate; OA, overall accuracy.

Taken together, asymptomatic infections elicit significantly weaker neutralizing antibody responses and the presence and abundance of neutralizing antibodies show strong correlation with anti-RBD and anti-S2 IgG binding antibodies.

### Sensitivity of Various Detection Schemes in Identifying AP

**Figure 4:**
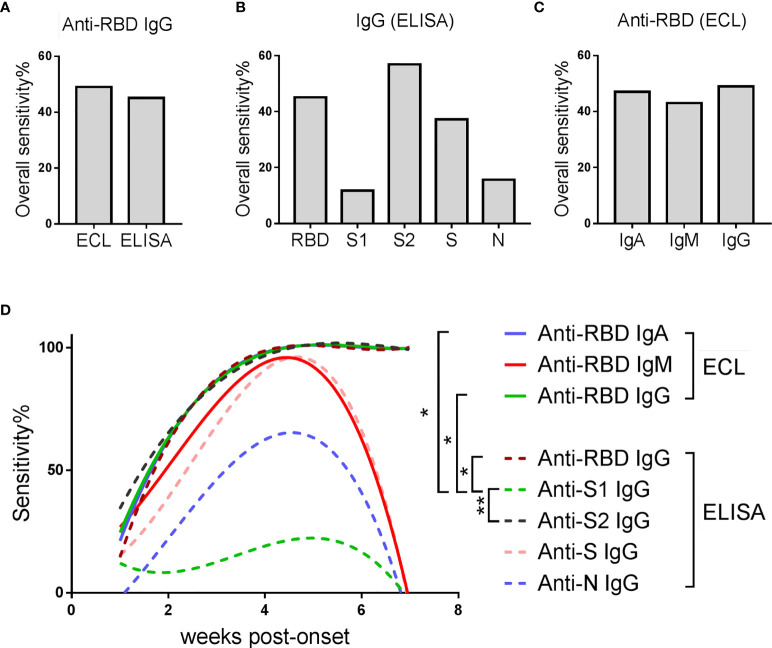
Comparison of sensitivity of various detection schemes in identifying AP. **(A)** Overall sensitivity of anti-RBD-IgG detected by different methods in all samples. **(B)** Overall sensitivity of IgG against different SARS-CoV-2 proteins detected by ELISA assay. **(C)** Overall sensitivity of different antibody isotypes against SARS-CoV-2-RBD detected by ECL assay. **(A–C)** Overall sensitivity = 100% × (the number of antibody positive samples/the number of all samples). **(D)** Sensitivity of various detection schemes over time. The line is a trend line fitted using the nonlinear regression method. Sensitivity = 100% × (the number of antibody positive samples at each time point/the number of total samples at each time point). Friedman test with Dunn's multiple comparisons test was used. A p-value of ≤ 0.05 was considered statistically significant (*p-values of ≤ 0.05; **p-values of ≤ 0.01).

In order to better control the spread of SARS-CoV-2, accurate identification of asymptomatic or pre-symptomatic infections is essential. We compared the overall sensitivity (the overall antibody positive rate in all samples covering all the time points) of different detection schemes in identifying asymptomatic infections (**Figures 4A–C**). By comparing the sensitivity of anti-RBD-IgG detected by different methods (**Figure 4A**), we found that the sensitivity of ECL was higher than that of ELISA (49.00% *vs.* 45.10%). By comparing the sensitivity of IgG against different SARS-CoV-2 proteins detected by ELISA (**Figure 4B**), we found that the detection of anti-S2-IgG can achieve the highest positive detection rate (56.86%). By comparing the sensitivity of different antibody isotypes against RBD detected by ECL (**Figure 4C**), we found that detection of IgG can achieve the highest sensitivity (49.00%).

In order to further explore whether and how the sensitivity of these schemes changes over time, they were plotted against the time post-onset (**Figure 4D**). The sensitivity of all schemes increased steadily in the first five weeks, and most of them declined rapidly afterwards, except anti-S2 IgG, anti-RBD IgG and anti-RBD IgA. Detection of anti-S2 IgG was better than anti-RBD IgG, manifested by a higher sensitivity in the first two weeks post-onset. Comparing the sensitivity-time curves of anti-RBD-IgG detected by ECL and ELISA, ECL showed slightly higher sensitivity than ELISA, mainly in the first 1-2 weeks after admission.

Taken together, detection of IgG against SARS-CoV-2-S2 protein by ECL may improve the sensitivity for early detection of asymptomatic infections.

## Discussion

In this study, we tested SARS-CoV-2-specific antibodies in 272 plasma samples from 59 COVID-19 patients. The dynamics of the different binding antibody isotypes against the major SARS-CoV-2 structural proteins, as well as neutralizing antibodies in patients with different disease severity were evaluated and the sensitivity of the different detection schemes were compared.

Since asymptomatic SARS-CoV-2 infection was reported, it has caused widespread concerns for not only the high risk of silent spread of the disease, but also limited understanding of the immune response (6). A previous study showed that asymptomatic individuals develop a weaker immune response to SARS-CoV-2 infection than symptomatic patients, including lower levels of virus-specific IgG (17). Consistent with this, we found that the overall antibody response is lower in asymptomatic infections than in symptomatic infections throughout the disease course, based on the response against various viral structural proteins, and the different isotypes of immunoglobulins against the RBD protein, as well as neutralizing antibodies. Besides, asymptomatic infections also show a lower antibody positive rate when compared with symptomatic infections at the same stage after infection, indicating that some asymptomatic infections develop antibody responses later. Taken together, these results demonstrate that asymptomatic infection induces a weaker humoral immune response.

We also found that the characteristics of antibody isotypes are different in patients with different disease severity. First, IgA appears later than IgG in asymptomatic infections, but earlier than IgG in symptomatic infections, especially in severe patients (**Figure 1C**). Our findings are consistent with the previous reports showing that IgA levels in severe cases were higher than those in mild or moderate cases (18, 19). Second, IgG is always dominant in asymptomatic individuals but not in symptomatic patients.

Neutralizing antibodies are important correlates of protection against infection. Detection of neutralizing antibodies in plasma is useful for evaluating the risk of infection in COVID-19 vaccinees and the risk of reinfection in COVID-19 convalescents (20). However, high-throughput detection of neutralizing antibodies is difficult due to the need for authentic SARS-CoV-2 viruses in biosafety level 3 laboratories and the long experimental procedure. Therefore, based on our findings, we propose here a candidate detection scheme to predict the level of neutralizing antibodies. Presence of IgG antibodies against S2 and RBD protein, which has the highest negative predictive value and the highest positive predictive value respectively, may best predict the presence of neutralizing antibodies. Further, the magnitude of neutralizing antibodies can be predicted from the anti-RBD IgG level. This approach may provide a safe alternative to predict the presence of neutralizing antibodies after infection.

Currently, a big challenge to control the pandemic is to rapidly and accurately identify asymptomatic infections at an early stage. As a gold standard, viral nucleic acid detection has high specificity, but its sensitivity is affected by many factors including sample collection. Serological testing can be a good supplement to nucleic acid testing (14). Although anti-RBD or anti-N IgG antibodies are often the major targets in serological testing, here we find that detection of IgG against the S2 protein by ECL improves the sensitivity for early identification of asymptomatic infections. Although the S2 protein of SARS-CoV-2 is relatively conserved within the beta-coronaviruses, no non-specific reactivity were observed in healthy donors (21), indicating that this test could be a good candidate for auxiliary testing.

In summary, our findings suggest that compared with a symptomatic infection, an asymptomatic infection elicits a weaker humoral immune response, that is dominated by an IgG-based response. Detection of IgG antibodies against S2 and RBD proteins may help to estimate the abundance of neutralizing antibodies. Additionally, the detection of IgG against SARS-CoV-2-S2 protein may be a good supplement to nucleic acid testing. Our findings should be further validated with a larger cohort and could only be applicable within the context of natural infection but not vaccination. In conclusion, this study provides a more sensitive antibody detection scheme for identification of asymptomatic infections that may help mitigate the COVID-19 pandemic.

## Data Availability Statement

The raw data supporting the conclusions of this article will be made available by the authors, without undue reservation.

## Ethics Statement

Ethical approval was obtained from the Ethics Committee of Guangzhou Eighth People’s Hospital (202002135). Written informed consent to participate in this study was provided by the participants’ legal guardian/next of kin.

## Author Contributions

XT, YL, AZ, BS, JincZ, and JingZ conceived the study. BL, LL, CW, and WC collected clinical specimen. ZC, AZ, PZ, JianZ, and SW executed the experiments. ZC, JianZ, FL, SL, DC, CW, WC, SW, YT, LD, PW, FC, JHY, JWY, and JF analyzed the data. ZC, JincZ, JingZ, and AZ drafted the manuscript. All authors contributed to the article and approved the submitted version.

## Funding

This work was supported by Emergency grants for prevention and control of SARS-CoV-2 from the Ministry of Science and Technology (2020YFC0841400), National Natural Science Foundation of China (82025001, 82072265), Guangdong Science and Technology Fund (2020B1111300001), Guangzhou Institute of Respiratory Health Open Project (Funds provided by China Evergrande Group, 2020GIRHHMS04), the 111 project (D18010), Zhong Nanshan Medical Foundation of Guangdong Province (ZNSA-2020001), Guangzhou Basic Research Program on People's Livelihood Science and Technology (202002020005). Guangzhou Institute of Respiratory Health Open Project (Funds provided by China Evergrande Group) (2020GIRHHMS04), and the Zhongnanshan Medical Foundation of Guangdong Province (ZNSA-2021005 and ZNSA-2020001).

## Conflict of Interest

The authors declare that the research was conducted in the absence of any commercial or financial relationships that could be construed as a potential conflict of interest.

## Publisher’s Note

All claims expressed in this article are solely those of the authors and do not necessarily represent those of their affiliated organizations, or those of the publisher, the editors and the reviewers. Any product that may be evaluated in this article, or claim that may be made by its manufacturer, is not guaranteed or endorsed by the publisher.

## Abbreviations

AP, asymptomatic patients; MP, mildly ill patients; SP, severely ill patients; HD, healthy donor; ECL, electrochemiluminescence; TPR, true positive rate; FPR, false positive rate; TNR, true negative rate; FNR, false negative rate; PPV, positive predictive value; NPV, negative predictive value; FDR, false discovery rate; OA, overall accuracy.

## References

[1] Ghaffari Darab M Keshavarz K Sadeghi E Shahmohamadi J Kavosi Z. The Economic Burden of Coronavirus Disease 2019 (COVID-19): Evidence From Iran. BMC Health Serv Res (2021) 21:132. 10.1186/s12913-021-06126-8 33573650 PMC7877330

[2] Do BN Tran TV Phan DT Nguyen HC Nguyen TTP Nguyen HC. Health Literacy, Ehealth Literacy, Adherence to Infection Prevention and Control Procedures, Lifestyle Changes, and Suspected COVID-19 Symptoms Among Health Care Workers During Lockdown: Online Survey. J Med Internet Res (2020) 22:e22894. 10.2196/22894 33122164 PMC7674138

[3] Fujita K Ito T Saito Z Kanai O Nakatani K Mio T. Impact of COVID-19 Pandemic on Lung Cancer Treatment Scheduling. Thorac Cancer (2020) 11:2983–6. 10.1111/1759-7714.13615 PMC7436133 32790028

[4] Chan JF Yuan S Kok KH To KK Chu H Yang J. A Familial Cluster of Pneumonia Associated With the 2019 Novel Coronavirus Indicating Person-to-Person Transmission: A Study of a Family Cluster. Lancet (2020) 395:514–23. 10.1016/S0140-6736(20)30154-9 PMC7159286 31986261

[5] Kam KQ Yung CF Cui L Tzer Pin Lin R Mak TM Maiwald M. A Well Infant With Coronavirus Disease 2019 With High Viral Load. Clin Infect Dis (2020) 71:847–9. 10.1093/cid/ciaa201 PMC7358675 32112082

[6] Kim SE Jeong HS Yu Y Shin SU Kim S Oh TH. Viral Kinetics of SARS-CoV-2 in Asymptomatic Carriers and Presymptomatic Patients. Int J Infect Dis (2020) 95:441–3. 10.1016/j.ijid.2020.04.083 PMC7196533 32376309

[7] Oran DP Topol EJ. The Proportion of SARS-CoV-2 Infections That Are Asymptomatic: A Systematic Review. Ann Intern Med (2021) 174:655–62. 10.7326/M20-6976 PMC7839426 33481642

[8] Babapoor-Farrokhran S Gill D Walker J Rasekhi RT Bozorgnia B Amanullah A. Myocardial Injury and COVID-19: Possible Mechanisms. Life Sci (2020) 253:117723. 10.1016/j.lfs.2020.117723 32360126 PMC7194533

[9] Bi Q Wu Y Mei S Ye C Zou X Zhang Z. Epidemiology and Transmission of COVID-19 in 391 Cases and 1286 of Their Close Contacts in Shenzhen, China: A Retrospective Cohort Study. Lancet Infect Dis (2020) 20:911–9. 10.1016/S1473-3099(20)30287-5 PMC7185944 32353347

[10] Pang J Wang MX Ang IYH Tan SHX Lewis RF Chen JI. Potential Rapid Diagnostics, Vaccine and Therapeutics for 2019 Novel Coronavirus (2019-Ncov): A Systematic Review. J Clin Med (2020) 9:623. 10.3390/jcm9030623 PMC7141113 32110875

[11] Lippi G Simundic AM Plebani M. Potential Preanalytical and Analytical Vulnerabilities in the Laboratory Diagnosis of Coronavirus Disease 2019 (COVID-19). Clin Chem Lab Med (2020) 58:1070–6. 10.1515/cclm-2020-0285 32172228

[12] Pan Y Long L Zhang D Yuan T Cui S Yang P. Potential False-Negative Nucleic Acid Testing Results for Severe Acute Respiratory Syndrome Coronavirus 2 From Thermal Inactivation of Samples With Low Viral Loads. Clin Chem (2020) 66:794–801. 10.1093/clinchem/hvaa091 32246822 PMC7184485

[13] Wang W Xu Y Gao R Lu R Han K Wu G. Detection of SARS-CoV-2 in Different Types of Clinical Specimens. JAMA (2020) 323:1843–4. 10.1001/jama.2020.3786 PMC7066521 32159775

[14] World Health Organization (2020). Available at: https://www.who.int/publications/i/item/diagnostic-testing-for-sars-cov-2.

[15] Huang Z Chen H Xue M Huang H Zheng P Luo W. Characteristics and Roles of Severe Acute Respiratory Syndrome Coronavirus 2-Specific Antibodies in Patients With Different Severities of Coronavirus 19. Clin Exp Immunol (2020) 202:210–9. 10.1111/cei.13500 PMC7405228 32706417

[16] Piccoli L Park YJ Tortorici MA Czudnochowski N Walls AC Beltramello M. Mapping Neutralizing and Immunodominant Sites on the SARS-CoV-2 Spike Receptor-Binding Domain by Structure-Guided High-Resolution Serology. Cell (2020) 183:1024–42.e21. 10.1016/j.cell.2020.09.037 32991844 PMC7494283

[17] Long QX Tang XJ Shi QL Li Q Deng HJ Yuan J. Clinical and Immunological Assessment of Asymptomatic SARS-CoV-2 Infections. Nat Med (2020) 26:1200–4. 10.1038/s41591-020-0965-6 32555424

[18] Yu HQ Sun BQ Fang ZF Zhao JC Liu XY Li YM. Distinct Features of SARS-CoV-2-Specific IgA Response in COVID-19 Patients. Eur Respir J (2020) 56:2001526. 10.1183/13993003.01526-2020 32398307 PMC7236821

[19] Ma H Zeng W He H Zhao D Jiang D Zhou P. Serum IgA, IgM, and IgG Responses in COVID-19. Cell Mol Immunol (2020) 17:773–5. 10.1038/s41423-020-0474-z PMC7331804 32467617

[20] Fagre AC Manhard J Adams R Eckley M Zhan S Lewis J. A Potent SARS-CoV-2 Neutralizing Human Monoclonal Antibody That Reduces Viral Burden and Disease Severity in Syrian Hamsters. Front Immunol (2020) 11:614256. 10.3389/fimmu.2020.614256 33391285 PMC7775388

[21] Wang Y Zhang L Sang L Ye F Ruan S Zhong B. Kinetics of Viral Load and Antibody Response in Relation to COVID-19 Severity. J Clin Invest (2020) 130:5235–44. 10.1172/JCI138759 PMC7524490 32634129

